# Cost-effectiveness of lifelong eculizumab versus disease monitoring of aHUS

**DOI:** 10.1093/ndt/gfaf166

**Published:** 2025-08-22

**Authors:** Giovany Orozco-Leal, Luke Vale, Yemi Oluboyede, Victoria Brocklebank, Andy Bryant, Tom Chadwick, Sarah Dunn, Sally Johnson, David Kavanagh, Ciara Kennedy, Jan Lecouturier, Chris Weetman, Edwin Wong, Len Woodward, Neil S Sheerin

**Affiliations:** Population and Health Sciences Institute, Newcastle University Newcastle upon Tyne, UK; Population and Health Sciences Institute, Newcastle University Newcastle upon Tyne, UK; Department of Health Services Research and Policy, London School of Hygiene and Tropical Medicine, London, UK; Putnam Associates LLC, Newcastle upon Tyne, UK; Population and Health Sciences Institute, Newcastle University Newcastle upon Tyne, UK; Population and Health Sciences Institute, Newcastle University Newcastle upon Tyne, UK; Population and Health Sciences Institute, Newcastle University Newcastle upon Tyne, UK; Population and Health Sciences Institute, Newcastle University Newcastle upon Tyne, UK; Translational and Clinical Research Institute, Newcastle University, Newcastle upon Tyne, UK; Population and Health Sciences Institute, Newcastle University Newcastle upon Tyne, UK; Translational and Clinical Research Institute, Newcastle University, Newcastle upon Tyne, UK; Population and Health Sciences Institute, Newcastle University Newcastle upon Tyne, UK; Middlesbrough Council, Middlesbrough, UK; Population and Health Sciences Institute, Newcastle University Newcastle upon Tyne, UK; Translational and Clinical Research Institute, Newcastle University, Newcastle upon Tyne, UK; Translational and Clinical Research Institute, Newcastle University, Newcastle upon Tyne, UK; aHUS Alliance Global Action, UK; Translational and Clinical Research Institute, Newcastle University, Newcastle upon Tyne, UK; aHUS Alliance Global Action, UK

**Keywords:** atypical haemolytic uraemic syndrome, chronic kidney disease, cost-effectiveness analysis, economic evaluation, kidney transplant

## Abstract

**Background:**

Atypical haemolytic uraemic syndrome (aHUS) is a rare but severe condition caused by complement dysregulation. Eculizumab prophylaxis prevents recurrence and improves survival, yet the benefits of lifelong treatment for patients are uncertain and the long-term costs for health services are substantial. This economic evaluation applied the results from the Stopping Eculizumab Treatment Safely in aHUS (SETS aHUS) trial and assessed the cost-effectiveness of replacing lifelong eculizumab with a disease monitoring strategy over the long term.

**Methods:**

A Markov model was used to estimate cost and quality-adjusted life years (QALYs) for a treatment withdrawal and disease monitoring strategy over the long term. Results from the SETS aHUS trial informed the risk of relapse diagnosis or progression, utility values and additional healthcare use. Time to relapse diagnosis was extrapolated using parametric survival functions.

**Results:**

The treatment withdrawal and disease monitoring strategy increased average patient QALYs by 0.08 [95% credible interval (CrI) −0.33–0.53], and reduced patient costs by £4 234 196 (95% CrI −£684 495 to −£6 403 694) compared with the lifelong delivery of eculizumab. The impact on survival estimates was low, as withdrawal patients had an average reduction of 0.0005 patient life years (LYs) (95% CrI −0.0029–0) over an 80-year time horizon. Withdrawal and monitoring had a 64% likelihood of being more effective and less costly than lifelong treatment. Results remained robust across multiple scenarios exploring uncertainties.

**Conclusion:**

Treatment withdrawal and disease monitoring was cost-effective compared with lifelong eculizumab therapy. Its adoption is expected to substantially reduce costs per patient and may improve average patient quality of life.

## Graphical Abstract

**Graphical Abstract:**
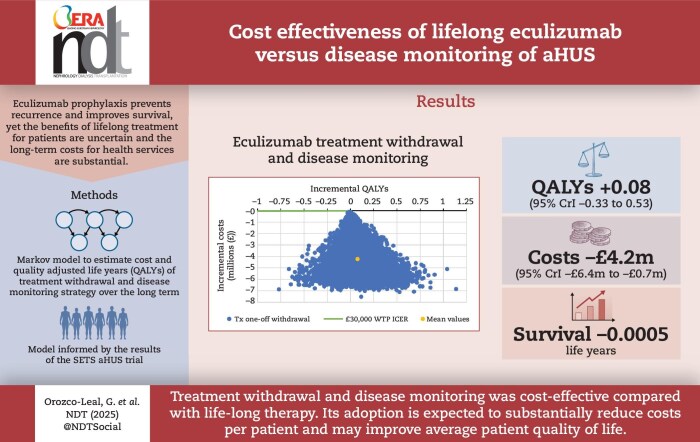

KEY LEARNING POINTS **What was known:**

- In 2015, the National Institute for Health and Care Excellence recommended eculizumab for the treatment of atypical haemolytic uraemic syndrome (aHUS) in the UK. The Stopping Eculizumab Treatment Safely in aHUS (SETS aHUS) study aimed to assess whether stopping treatment with a disease monitoring protocol could be a safe and cost-effective alternative to lifelong treatment.

**This study adds:**

- Using a Markov model based on the SET aHUS data, we estimated the cost-effectiveness of stopping eculizumab treatment with a disease monitoring strategy in eligible patients over their lifetime. Eculizumab withdrawal and disease monitoring was highly likely to be a cost-effective treatment.

**Potential impact:**

- Stopping eculizumab treatment with a disease monitoring strategy would save health services considerable resources per patient (decreasing costs on average by >40% versus the cost of lifelong treatment). The impact on survival is minimal, while the impact on quality of life is small and positive on average.

## INTRODUCTION

Atypical haemolytic uraemic syndrome (aHUS) is a rare, life-threatening disease characterised by a severe inflammation of blood vessels and the formation of blood clots throughout the body, leading to tissue injury and subsequently thrombotic microangiopathy (TMA). The disease more often manifests clinically as TMA involving the kidneys and if left untreated can lead to kidney failure [1, 2].

In the UK, the incidence of aHUS is 0.4–0.5 cases per million per year. In 70% of these cases the disease is associated with excessive activation of the complement system due to a genetic or acquired abnormality [2]. Eculizumab is part of a group of drugs known as C5 inhibitors; C5 being an important protein involved in complement activation. Thus eculizumab prevents tissue damage from excessive activation of the complement system [2].

In 2015, the National Institute for Health and Care Excellence (NICE) recommended eculizumab for the treatment of aHUS [2]. NICE recommended that treatment be lifelong but also recommended research to investigate the safety of dose adjustments and treatment discontinuation [2]. The introduction of eculizumab changed the treatment pathway and improved the prognosis of aHUS patients, but treatment duration is still debated, with a dearth of studies investigating the optimal duration of treatment [3]. Treatment with eculizumab is not without risk, as its delivery is associated with an increased risk of meningococcal infection [4].

The most salient issue stimulating the debate on treatment duration with eculizumab is its prohibitive price, as lifelong treatment duration comes with a substantial cost to health services and patients, even in countries where treatment is free at the point of use, due to the frequency of hospital appointments that patients must attend [3, 5].

The Stopping Eculizumab Treatment Safely in aHUS (SETS aHUS) trial came about in response to NICE's call for a study assessing the safety of stopping eculizumab treatment [1, 2]. The SETS aHUS trial was a stage 2, single-arm, non-blinded assessment of the safety and cost-effectiveness of eculizumab withdrawal in patients with aHUS using a Bayesian factor single-arm design [6]. Due to the trial methodology and the small sample size (*n* = 28), a within-trial economic evaluation was not deemed appropriate. Instead, the trial collected costs and health outcomes for the trial sample and for a comparison group of patients under treatment maintenance. The purpose of this data collection was to provide an assessment of the cost-effectiveness of eculizumab withdrawal with a disease monitoring protocol over the long term.

In accordance with the National Institute for Health and Care Research threaded publication model [7], this article complements the respective publications of the clinical outputs analysis [8] and the qualitative analysis exploring participant views of the withdrawal protocol. This article reports how we used clinical and health economic data from the SETS aHUS trial along with data from the published literature to construct an economic model to assess the cost-effectiveness of substituting lifelong therapy with a system of protocolised surveillance and a strategy for treatment reintroduction and compared it with the lifelong delivery of eculizumab.

## MATERIALS AND METHODS

Full details on the methodology used for the economic analysis are in the health economic analysis plan (Appendix 1). Further details of the trial protocol are reported in Dunn *et al.* [1] and its primary results in Bryant *et al.* [8].

### Population

The target population was adult patients diagnosed with aHUS receiving eculizumab to treat the disease in native kidneys with either normal renal function (CKD stage 0) or chronic kidney disease stages 1–3 (CKD 1–3). Patients undergoing transplant, or patients at or below CKD stage 4 were not included. In the base-case analysis, a cohort of patients 20 years of age, comprising 51% males and 49% females was modelled based on the trial sample and treatment maintenance cohort of the SETS aHUS trial [8].

### Setting and location

The setting was the UK National Health Service (NHS). After eculizumab initiation, patients maintaining treatment were eligible to have it delivered at home or at the hospital by a specialist nurse. Patients under treatment withdrawal attended inpatient visits at the hospital to assess their disease status. The restart of treatment was also delivered in the inpatient setting.

### Intervention and comparator

Following the trial protocol, patients in all arms of the model had completed at least 6 months of eculizumab treatment, including vaccination against meningococcal infections and other antibiotic prophylaxis [1]. At the start of the model, patients in the withdrawal arm ceased eculizumab treatment and commenced disease monitoring (consistent with the SETS aHUS trial protocol [1]) for the rest of their lives or until treatment was restarted. If treatment was restarted it was maintained for the rest of the patient lifetime. Patients in the comparison arm of the model remained on treatment with eculizumab for the rest of their lives.

### Model structure

To inform the model structure, a literature review was conducted in June 2023 using Embase and Web of Science to identify model-based economic evaluations on aHUS. Three economic evaluations were identified [9–11], of which two were model-based analyses [9, 10] in addition to technology appraisals conducted by NICE (HST1 and TA710) [12, 13].

**Figure 1:**
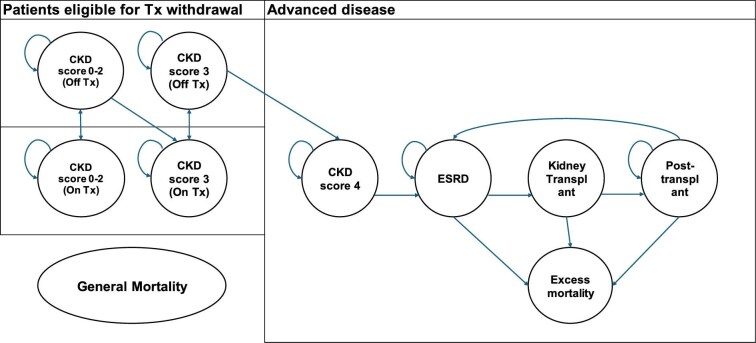
Economic model structure. Tx: treatment with eculizumab.

The *de novo* economic model was built in Excel 2016 (Microsoft, Redmond, WA, USA) (Fig. 1). A Markov model structure based on the most recent submission to NICE was considered the best approach [12], informed by data from the SETS aHUS trial. Health states in the model represent CKD stages based on estimated glomerular filtration rate (eGFR) scores, from normal kidney function (eGFR ≥90 ml/min/1.73 m^2^, CKD stage 1) to end-stage renal disease (ESRD; kidney failure with eGFR <15 ml/min/1.73 m^2^) [14]. The first health state grouped patients with CKD stages 0–2, the next health states were CKD 3, CKD 4, ESRD, kidney transplant and a post-transplant state. Patients entered the model at either CKD 0–2 or CKD 3, based on the disease distribution in the SETS aHUS trial. CKD 0–2 patients could stay off treatment, restart treatment, progress to CKD 3 and restart treatment or move to the death state. CKD 3 patients could stay off treatment, restart treatment at CKD 3 disease, progress to CKD 4 and restart treatment or move to the death state. Patients at CKD 0–2 and CKD 3 receiving treatment were assumed to stay in their CKD health state for the rest of the time horizon.

All patients progressing to CKD stage ≥4 were assumed to continue to receive eculizumab treatment and could stay in CKD 4 until death or disease progression to ESRD. When patients progressed to ESRD they could stay in ESRD or receive a kidney transplant, which is a tunnel state to the post-transplant state. Patients progressing to ESRD or beyond faced a higher risk of mortality due to disease severity and transplant-related causes. The model structure is presented in Fig. 1.

A cycle length of 2 weeks and a half-cycle correction was used to capture treatment cycles and disease stages. A lifetime horizon was considered appropriate for the base-case analysis, ending the simulation when surviving patients were 100 years old.

### Health state transition probabilities

**Figure 2:**
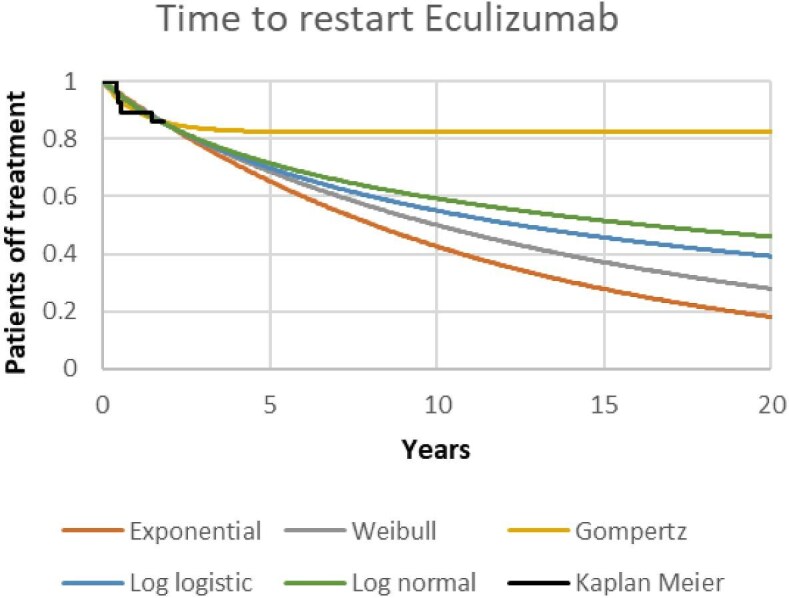
Model predictions of time to aHUS relapse diagnosis.

Disease distribution at baseline based on CKD stages was 96% (*n* = 27) at CKD 0–2 and 4% at CKD 3 (*n* = 1), corresponding to the baseline distribution in the SETS aHUS trial [8]. Time-to-event data from the trial showed four patients (14%) had a relapse diagnosis and restarted treatment after 2 years of eculizumab withdrawal, of which one patient had a primary outcome event with a persistent >20% decrease in eGFR, while the rest recovered their kidney function after restarting treatment [8]. The probability of disease relapse over time was derived from fitting standard parametric functions to the Kaplan–Meier data [15]. Due to the small sample size, the functions fitted included exponential, Weibull, Gompertz, log logistic and lognormal (Fig. 2) [15]. The risk of disease progression from aHUS relapse was assumed constant over time. This is potentially a conservative assumption, since further data from the aHUS SETS trial cohort showed that the patients who experienced a primary outcome event recovered after the study follow-up period [8].

The differences in Bayesian information criterion (BIC) scores, used to measure the goodness of fit to the data, were small across the five functions. The exponential function was judged as having the best BIC score; however, the lognormal function was selected for the base case due to its decreasing hazard over time, in line with evidence from Nikolaidis *et al.* [16]; moreover, its long-term predictions were a midpoint between the predictions considered (Fig. 2).

Patients at CKD 0–2 and CKD 3 who are receiving or restarting treatment with eculizumab are assumed to have no risk of progression. For patients at CKD stage 4, the risk of progressing to ESRD was based on the results from Rondeau *et al.* [17].

Patients face a mortality risk sourced from Office of National Statistics life tables for the general UK population. Patients progressing to the health states of ESRD, transplant and post-transplant care face an additional 0.4% excess mortality rate per cycle based on the 5% 6-month excess mortality used in NICE TA710 [18].

### Analysis of health economics trial data

The SETS aHUS trial collected quality of life data using the EuroQol five-dimension, five-level (EQ-5D-5L) questionnaire and healthcare resource use from disease management using healthcare utilisation questionnaires at baseline and months 1, 3, 6, 9, 12, 18 and 24 of follow-up. Data were collected for the trial population (*n* = 28) and a comparison cohort (*n* = 11) maintaining eculizumab from trial baseline and across each follow-up time point.

Missing data were a primary issue during data analysis, as a complete case analysis of total quality-adjusted life years (QALYs) showed that 61% of participants had at least one missing EQ-5D-5L in the withdrawal group (11 complete records) and 91% of participants had at least one missing EQ-5D-5L in the comparison group (1 complete record). Similar levels of missingness were observed in the total healthcare use data, with 57% missing at least one questionnaire in the withdrawal group (12 complete records) and 91% missing at least one questionnaire in the comparison group (1 complete record). Complete case estimates were presented descriptively as mean total QALYs and mean total costs for each treatment arm.

**Figure 3:**
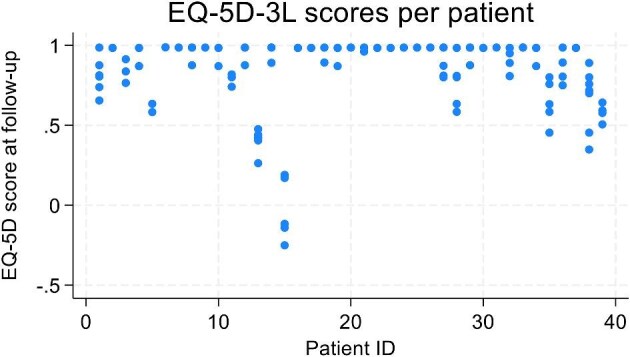
Mapped EQ-5D-3L scores per patient from the SETS aHUS trial.

**Figure 4:**
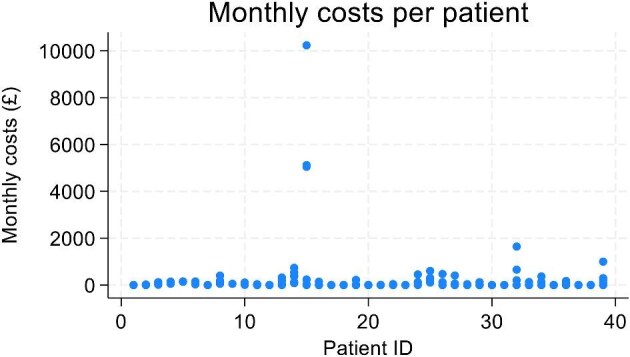
Monthly costs per patient from the SETS aHUS trial.

Due to the small sample size in the trial and the large proportion of missing data, available case analysis was deemed more appropriate to inform the utility changes for withdrawing from treatment and disease relapse [19]. A multivariate random effects model was used instead, controlling for time at follow-up, baseline utility, age and gender. Monthly healthcare resource use data were also assessed using available case analysis, with a random effects model controlling for time, baseline costs, age and gender. Statistical analyses were performed using Stata version 18 (StataCorp, College Station, TX, USA) and figures drawn using Microsoft Excel 2016. The EQ-5D-5L and resource use data of one patient were considered as outliers (see Figs. 3 and 4). Since the small sample size makes the analysis vulnerable to outlier effects, the scenario analysis includes the impact of controlling for the outlier on costs and QALYs.

### Health outcomes

Health outcomes in the model were measured using QALYs. Quality-of-life weights were calculated based on patient responses to the EQ-5D-5L questionnaire from the SETS aHUS trial [1, 20]. Responses were transformed into EQ-5D-3L scores using UK population values adjusted by age and gender [21, 22].

Data from the aHUS trial was used to inform quality of life in the CKD 0–2 and CKD 3 health states, and utility changes from treatment withdrawal and disease relapse. For more advanced disease stages not covered by the 2-year trial follow-up (CKD stage ≥4), quality of life values were sourced from the published literature and the technology assessments approved by NICE on aHUS [9, 10, 12, 13]. A scenario analysis assessed the impact of using quality-of-life weights from the literature rather than the trial values.

### Resource use and costs

All costs were presented using 2022 British pounds Sterling (£ GBP). Treatment costs with eculizumab and later ravulizumab were sourced from the British National Formulary, including treatment initiation and treatment maintenance schedules [23]. Delivery costs beyond drug acquisition were sourced from the trial for the hospital setting since delivery costs in the homecare setting are currently funded by Alexion Pharma UK [24].

The resource use of disease monitoring during both treatment maintenance and withdrawal were sourced from NICE TA710 and cost values were updated for inflation to 2022 values [25]. Disease management costs at CKD stage 4 and ESRD were also sourced from NICE TA710, while kidney transplant and post-transplantation costs were sourced from their respective NHS tariffs, with additional healthcare staff costs sourced from Jones et al. [25–27].

Data from the SETS aHUS trial was used to inform disease management costs for CKD 0–2 and CKD 3 patients withdrawing from eculizumab and for the comparison group maintaining treatment [1]. Cost data in the SETS aHUS trial healthcare utilisation questionnaires were used to inform additional healthcare costs from treatment maintenance, withdrawal and disease relapse in the model [1].

### Perspective

Costs were included from an NHS and personal social services perspective. Outcomes were self-reported by patients through questionnaires measuring changes in health-related quality of life. The economic analysis was carried out using a lifetime horizon, with an annual discount rate on all outputs of 3.5% [28].

### Model-based analysis

Cost-utility analyses were conducted at the cohort level and presented in an incremental analysis over a patient's lifetime. A deterministic sensitivity analysis was used to identify the parameters with the largest impact on cost-effectiveness and a probabilistic analysis was conducted using the parametric distribution considered to best estimate the uncertainty around each input parameter [29] (e.g. the Gamma distribution primarily for cost parameters, the Beta distribution for probabilities and proportions; see Table 1 for more details). When estimates of variation were not available, a 10% standard error around the mean was assumed.

**Table 1:** Model parameters.

Parameter	Mean or %	SE	PSA distribution	Source
Starting age (years)	20			Assumption
Males	51%			[8]
Disease distribution				
% of CKD 0–2	96%	9.6%	Beta	[8]
% of CKD 3	4%			[8]
Disease progression after relapse				
No treatment				
CKD 0–2 to CKD 3	25%	2.5%	Beta	[8]
CKD 3 to CKD 4	25%	2.5%	Beta	[8]
On treatment				
CKD 0–2 to CKD 3	0			Assumption
CKD 3 to CKD 4	0			Assumption
Disease progression to advanced disease per cycle				
CKD 4 to ESRD	2.55%	0.25%	Beta	[25]
ESRD to kidney transplant	3.25%	0.32%	Beta	[2]
Transplant failure	11.64%	2.72%	Lognormal	[5]
Excess mortality per cycle				
ESRD	0.41%	0.04%	Normal	[25]
Transplant	0.41%	0.04%	Normal	Assumption
Post-transplant	0.41%	0.04%	Normal	Assumption
Utility values				
Baseline CKD 0–3	0.86	0.03	Beta	[8]
Transplant procedure	0.66	0.07	Beta	[2]
Disutilities				
Treatment withdrawal	0.01	0.02	Normal	[8]
Disease relapse	−0.02	0.04	Normal	[8]
CKD 4	−0.15	0.02	Normal	[25]
ESRD	−0.21	0.02	Normal	[25]
Post-transplant	−0.21	0.02	Normal	[25]
Drug acquisition costs				
Eculizumab	£3150			[23]
Ravulizumab	£16 621			[23]
% Home delivery	71%	7.1%	Beta	SETS aHUS nurses [8]
Hospital drug delivery	£1429	142.90	Gamma	SETS aHUS nurses [8]
Antibiotic medications	£7	2.59	Gamma	[30]
Disease monitoring costs per cycle				
Maintenance	£102	10.24	Gamma	[25, 27]
Withdrawal month 1	£424	42.37	Gamma	[25, 29]
Withdrawal months 2–8	£215	21.53	Gamma	[25, 27]
Withdrawal month >8	£111	11.11	Gamma	[25, 27]
Disease management costs per cycle				
Treatment withdrawal	−£32	62	Gamma	[8, 26]
Disease relapse	−£150	165	Gamma	[8, 26]
CKD 4	£18	1.77	Gamma	[25]
ESRD	£24	2.36	Gamma	[25]
Kidney transplant	£16 037	1 564	Gamma	[25, 26]
Post-transplant	£306	25	Gamma	[25, 26]

PSA: probabilistic sensitivity analysis.

The scenario analysis explored the impact on cost-effectiveness estimates from an optimistic and pessimistic risk to restart treatment over time using the Gompertz function and exponential distribution, respectively; replacing the quality-of-life weights from the trial for values sourced from the literature; controlling for a patient identified as an extreme outlier in quality of life and resource use data from the trial and reducing the price of eculizumab by 50%.

We also explored a scenario comparing lifelong treatment with an alternative to the current treatment withdrawal strategy, where patients who relapse receive a 3-month course of eculizumab before stopping treatment again, based on some of the patterns observer by Wijnsma *et al.* [3]. In this scenario, patients can restart treatment as many times as they need rather than maintaining lifelong treatment after the first relapse. As this strategy was not assessed within the main SETS aHUS trial, clinical effectiveness in this scenario was based on the assumptions that the risk of a future relapse was the same as the risk of the first relapse and that patients on treatment were at no risk of CKD stage deterioration.

The 2015 guidelines on the standard care of aHUS were updated in 2021 to include ravulizumab as an alternative C5 inhibitor in the treatment pathway [25]. As ravulizumab was not used during the SETS aHUS study, a scenario analysis explored a comparison of lifelong treatment versus treatment withdrawal and monitoring using ravulizumab instead of eculizumab to treat aHUS. This followed the assumption of clinical equivalence between eculizumab and ravulizumab in NICE TA710 [12, 25].

Table 1 summarises the parameters used to inform the base-case economic model.

## RESULTS

### Trial-based cost-effectiveness results

#### Trial-based quality of life and treatment effects

Total QALYs for the SETS aHUS trial population withdrawing from eculizumab with 11 complete records was 1.60 [standard error (SE) = 0.18] over 2 years of follow-up. For the comparison arm maintaining treatment with only one complete record, total QALYs was 1.94 (no SE calculable) over 2 years. Results from the random effects model showed a positive but non-statistically significant difference in utility values for the withdrawal arm (0.006, *P* = .70) and a negative but non-statistically significant difference from diagnosed relapse (−0.015, *P* = .70; Table 2).

**Table 2:** Random effects results for EQ-5D-3L utility scores—SETS aHUS trial.

Variable	Coefficient	SE	*P*-value	95% CI
Month	0.002	0.001	.04	0.000–0.003
Age	0.001	0.001	.33	−0.001–0.002
Male	−0.016	0.025	.52	−0.065–0.033
Baseline utility	0.959	0.059	.00	0.843–1.075
Tx withdrawal	0.006	0.016	.70	−0.026–0.038
Relapse	−0.015	0.040	.70	−0.093–0.063
Constant	0.028	0.056	.62	−0.082–0.138

CI: confidence interval; Tx: treatment.

#### Trial-based resource use

Total cost per patient for the SETS aHUS trial population withdrawing from eculizumab with 21 complete records was £5883 (SE = 4205) over 2 years of follow-up. For the comparison arm maintaining treatment with only four complete records, total cost was £352 (SE = 228) over 2 years. Results from the linear random effects model showed a negative but non-statistically significant difference in average monthly costs for the withdrawal cohort (−£70, *P* = .60) and a negative but non-statistically significant difference from diagnosed relapse (−£325, *P* = .36; Table 3).

**Table 3:** Random effects results for monthly healthcare utilisation—SETS aHUS trial.

Variable	Coefficient	SE	*P*-value	95% CI
Month	10	7	0.13	−3–23
Age	−3	4	0.48	−11–5
Male	241	149	0.11	−52–533
Baseline costs	0.38	0.15	0.01	0.08–0.68
Tx withdrawal	−70	133	0.60	−332–191
Relapse	−325	358	0.36	−1025–376
Constant	−6	160	0.97	−320–309

CI: confidence interval; Tx: treatment.

### Decision model results

Table 4 shows the deterministic and probabilistic cost-effectiveness results of the base-case analysis. Probabilistic results showed that stopping eculizumab with a disease monitoring strategy led to average cost-savings of −£4 234 196 [incremental costs 95% credible interval (CrI) −£684 495 to −£6 403 694] per patient and an increase in QALYs of 0.08 (incremental QALYs 95% CrI 0.53 to −0.33 QALYs) compared with lifelong maintenance of eculizumab. Stopping eculizumab also led to a reduction in survival of −0.0005 LYs (incremental LYs 95% CrI 0 to −0.0029).

**Table 4:** Base-case model results.

Treatment arm	LYs	QALYs	Costs	Incremental QALYs	Incremental costs	ICER
Deterministic base case						
Maintenance	25.44	21.06	£8 880 187			
Withdrawal	25.44	21.14	£4 301 807	0.08	−£4 578 380	Withdrawal dominant
Probabilistic base case						
Maintenance	25.44	21.04	£8 879 821			
Withdrawal	25.44	21.12	£4 645 625	0.08	−£4 234 196	Withdrawal dominant

ICER: incremental cost-effectiveness ratio.

### Effects of uncertainty

**Figure 5:**
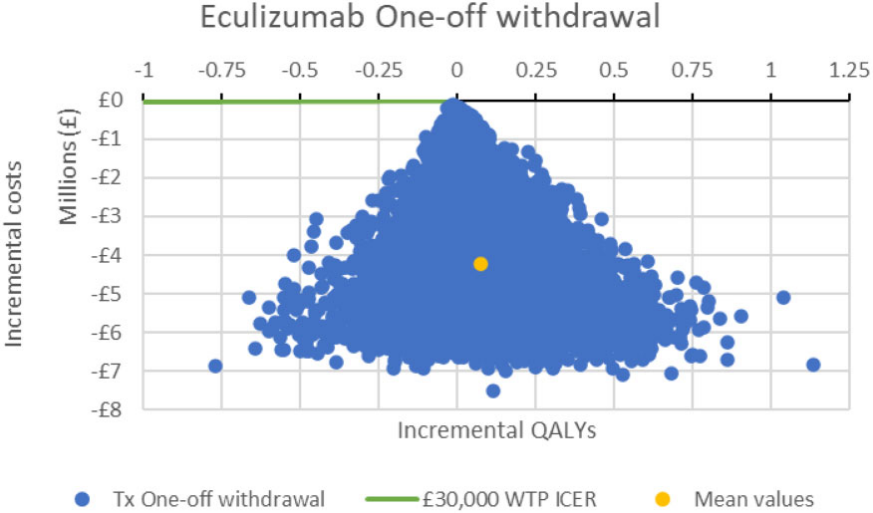
Cost-effectiveness plane. ICER: incremental cost-effectiveness ratio; Tx: treatment.

**Figure 6:**
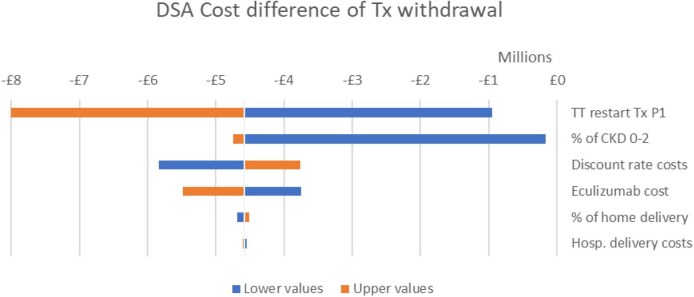
Deterministic sensitivity analysis on the cost difference of treatment withdrawal. DSA: deterministic sensitivity analysis; TT: time to treatment; Tx: treatment.

**Figure 7:**
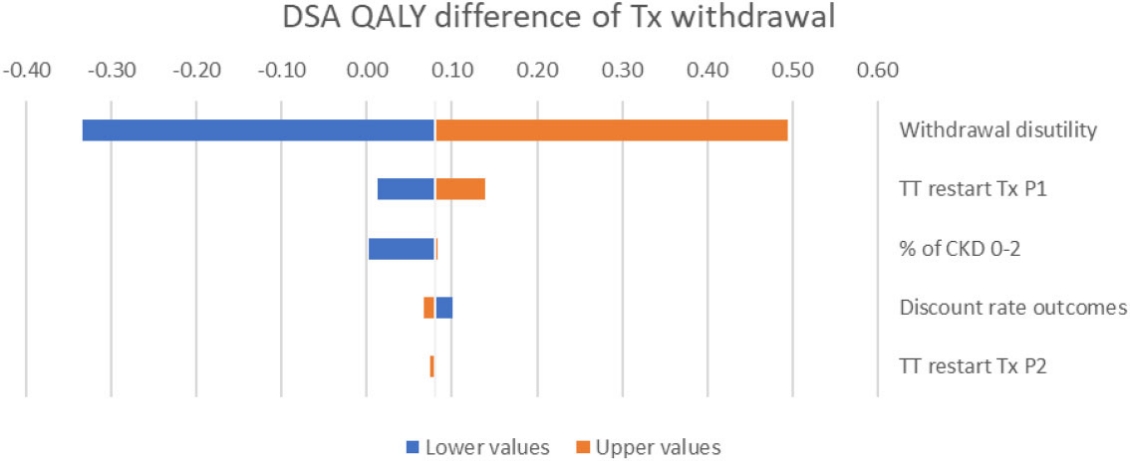
Deterministic sensitivity analysis on the QALY difference of treatment withdrawal. DSA: deterministic sensitivity analysis; TT: time to treatment; Tx: treatment.

**Figure 8:**
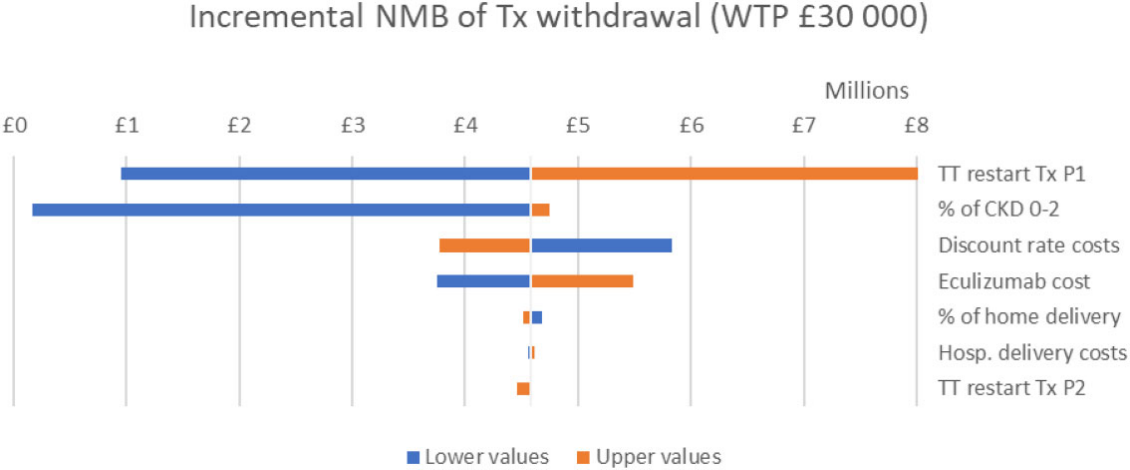
Deterministic sensitivity analysis for the incremental net monetary benefit of treatment withdrawal. NMB: incremental net monetary benefit; TT; time to treatment; Tx; treatment; WTP: willingness to pay.

Results from the probabilistic sensitivity analysis showed that a treatment withdrawal strategy had a 100% probability of being cost-effective over all values for society's willingness to pay for a QALY up to £250 000 and was more effective and less costly in 64% of the simulations (Fig. 5). A one-way sensitivity analysis on the deterministic incremental net monetary benefit (NMB) showed that the most impactful parameters on the cost-effectiveness results were the risk of a diagnosed relapse after withdrawal, the initial proportion of CKD 0–2 patients versus more severe CKD stages at withdrawal and the cost of eculizumab. The most impactful parameter for quality of life was the disutility of eculizumab withdrawal. The incremental NMB of eculizumab withdrawal was £4 580 783 for a £30 000 per QALY threshold. Results are presented in Figs. 6–8.

The scenario analysis showed that a strategy allowing for multiple withdrawals after reinitiation, the use of trial utility estimates after controlling for an outlier and the risk of a relapse diagnosis over time had the biggest impact on effectiveness, while halving the price of eculizumab, the risk of reinitiation over time and the multiple withdrawal strategy had the largest effect on costs (Table 5).

**Table 5:** Scenario analysis.

Scenarios	LYs	QALYs	Costs	Incremental QALYs	Incremental costs	ICER
Deterministic base case						
Maintenance	25.44	21.06	£8 880 187			
Withdrawal	25.44	21.14	£4 301 807	0.08	−£4 578 380	Withdrawal dominant
Multiple withdrawals						
Maintenance	25.44	21.06	£8 880 187			
Withdrawal	25.44	21.21	£316 412	0.15	−£8 563 775	Withdrawal dominant
Gompertz distribution Tx restart (favourable)						
Maintenance	25.44	21.06	£8 880 187			
Withdrawal	25.44	21.18	£1 704 273	0.12	−£7 75 914	Withdrawal dominant
Exponential distribution Tx restart (unfavourable)						
Maintenance	25.44	21.06	£8 880 187			
Withdrawal	25.44	21.11	£6 028 386	0.05	−£2 851 801	Withdrawal dominant
Baseline QALYs from the literature						
Maintenance	25.44	24.31	£8 880 187			
Withdrawal	25.44	24.39	£4 301 807	0.08	−£4 578 380	Withdrawal dominant
Baseline QALYs controlling for an extreme outlier						
Maintenance	25.44	21.56	£8 880 187			
Withdrawal	25.44	21.69	£4 301 807	0.13	−£4 578 380	Withdrawal dominant
Direct costs controlling for an extreme outlier						
Maintenance	25.44	21.06	£8 880 187			
Withdrawal	25.44	21.14	£4 305 917	0.08	−£4 574 269	Withdrawal dominant
Ravulizumab treatment						
Maintenance	25.44	21.06	£8 585 465			
Withdrawal	25.44	21.14	£4 167 673	0.08	−£4 417 793	Withdrawal dominant
2-year time horizon						
Maintenance	1.963	1.696	£841 835			
Withdrawal	1.963	1.707	£234 985	0.01	−£606 850	Withdrawal dominant
10-year time horizon						
Maintenance	8.58	7.383	£3 107 300			
Withdrawal	8.58	7.422	£928 246	0.04	−£2 179 054	Withdrawal dominant
50% eculizumab price reduction						
Maintenance	25.44	21.06	£4 616 089			
Withdrawal	25.44	21.14	£2 249 891	0.08	−£2 366 198	Withdrawal dominant

ICER: incremental cost-effectiveness ratio.

## DISCUSSION

The aim of this economic analysis was to assess the cost-effectiveness of using a treatment stoppage strategy on aHUS patients with CKD stage 0–3 as an alternative to a lifelong treatment eculizumab maintenance strategy. Data from the trial on patient quality of life, resource use and clinical safety of eculizumab withdrawal were used to inform an economic model assessing the long-term cost-effectiveness impact for each patient.

The base-case analysis showed that a treatment withdrawal strategy with disease monitoring has the potential to improve patient quality of life on average by 0.08 QALYs and reduce lifetime costs by £4 234 196 compared with the lifetime maintenance of eculizumab. Model inputs included the risks of treatment reinitiation and disease progression after reinitiation, where the treatment withdrawal strategy led to a small average reduction in length of life of 0.0005 LYs over an 80-year time horizon (equivalent to 4 h over a lifetime). The withdrawal and monitoring strategy was on average cost-saving, with a 64% probability of being more effective and less costly than treatment maintenance. The deterministic analysis identified the risk of relapse, the initial proportion of patients with CKD 0–2 versus CKD 3 and the cost of eculizumab as the primary drivers of cost-effectiveness.

Although previous models have attempted to include treatment withdrawal and restart [9, 12], our model is the first to use trial data to inform the time from withdrawal to restarting treatment using the SETS aHUS trial results [1]. This further showed that models relying on an assumption of a constant hazard over time for restarting treatment are potentially pessimistic, while using parametric models allowed us to relax this assumption.

Our analysis further explored scenarios that included changing the C5 inhibitor used to treat aHUS to ravulizumab or changing the re-treatment strategy to allow further withdrawals. Moreover, although the withdrawal and disease monitoring strategy remained cost-effective compared with lifelong treatment, due to the scarcity of data available these analyses relied on strong assumptions. Thus these results indicate a potential for further research, as we could expect the cost of such research to be more than offset by cost-savings in the use eculizumab or ravulizumab.

The primary limitations of the economic analysis stemmed from the small sample size in the SETS aHUS trial (given that aHUS is a rare disease), compounded by large proportions of missing data and the effects of extreme outliers. Moreover, the follow-up time of the SETS aHUS trial (approximately 2 years after stopping treatment) was significantly shorter compared with the scope of the model (lifetime of a 20-year-old patient). Nevertheless, our analysis suggested likely gains in quality of life and evidence of cost-savings over the first 2 and 10 years of treatment withdrawal (Table 5). Our analyses further incorporated the imprecision in estimates caused by these uncertainties, and it is reassuring that our sensitivity analyses confirmed the conclusions drawn from the study, additional analyses on the impact of treatment with C5 inhibitors increasing meningococcal infection risks were not performed, given the absence of data from the trials, but were expected to reinforce our conclusions; moreover there is potential for the use of real-world evidence to further strengthen the evidence available.

## CONCLUSIONS

The long-term benefits of lifelong treatment with eculizumab are still uncertain. Results from our model-based cost-effectiveness analysis suggest that eculizumab withdrawal coupled with disease monitoring for eligible aHUS patients will result in considerable cost-savings and a potential improvement in patient quality of life, with very little impact on survival. Consequently, treatment withdrawal is highly likely to be cost-effective. This conclusion remained robust to the wide range of scenarios considered.

## Data Availability

The data underlying this article are available in the article.
